# Effects of 8 Weeks with Embodied Learning on 5–6-Year-Old Danish Children’s Pre-reading Skills and Word Reading Skills: the PLAYMORE Project, DK

**DOI:** 10.1007/s10648-022-09671-8

**Published:** 2022-04-14

**Authors:** Linn Damsgaard, Anne-Mette Veber Nielsen, Anne Kær Gejl, Anne Sofie Bøgh Malling, Søren Kildahl Jensen, Jacob Wienecke

**Affiliations:** 1 Department of Nutrition Exercise and Sports, University of Copenhagen, Copenhagen, Denmark; 2 The National Centre for Reading, University College Copenhagen, Copenhagen, Denmark; 3 Department of Sports Science and Clinical Biomechanics, University of Southern Denmark, Odense, Denmark; 4 Department of Sport and Social Sciences, Norwegian School of Sport Sciences, Oslo, Norway

## Abstract

The aim of this study was to investigate the effects of embodied learning on children’s pre-reading and word reading skills. We conducted a three-armed randomized controlled trial including two intervention groups and one control group. One hundred forty-nine children from grade 0 (5–6 years old) who had just started school were recruited from 10 different classes from four elementary schools. Within each class, children were randomly assigned to receive teaching of letter-sound couplings and word decoding either with whole-body movements (WM), hand movements (HM), or no movements (CON) over an 8-week period. Children were evaluated on pre-reading, word reading, and motor skills before (T1), immediately after (T2), and after 17–22 weeks of retention period (T3) following the intervention. Between-group analysis showed a significant improvement in children’s ability to name letter-sounds correctly from T1 to T2 (*p* < 0.001) and from T1 to T3 (*p* < 0.05) for WM compared to CON*.* HM and WM improved significantly in naming conditional letter-sounds from T1 to T2 (*p* < 0.01, *p* < 0.01) compared to CON and from T1 to T3 for the HM group compared to CON (*p* < 0.05). We did not find an effect on word reading or a correlation between motor skill performance and reading. Results from the present study suggest that there are beneficial effects of using whole-body movements for children. Hand motor movements indeed also had a performance effect on letter-sound knowledge; however, the whole-body movements had longer-lasting effects. We do not see an effect on whole word reading.

## Introduction

Reading is a complex unique human skill, which serves an essential role in modern society. Poor spelling and reading skills in children and adolescents have been associated with poor academic achievement (Savolainen et al., 2008; Smart et al., 2017; Willcutt et al., 2007), school dropout (Daniel et al., 2006; McGee et al., 2002), and lower occupational status in adulthood (Savolainen et al., 2008). The acquisition and development of competent reading skills in childhood are therefore critical to functioning and well-being later in life.

Learning to read in alphabetic orthographies requires children to learn and remember the connections between phonemes of spoken words and graphemes of written words (letter-sound knowledge) as these connections are essential for the decoding of unfamiliar words (Byrne & Fielding-Barnsley, 1989). Phoneme awareness and letter knowledge measured before the outset of formal reading instruction are unique predictors of later reading and spelling abilities (Caravolas et al., 2012; Furnes & Samuelsson, 2009; Hulme et al., 2012; Kirby et al., 2008; Melby-Lervåg et al., 2012; Schatschneider et al., 2004). Danish may be particularly challenging for learners as it has an irregular orthography including standard and conditional pronunciations of letters with some similarities to English (Elbro, 2014; Juul & Sigurdsson, 2005). Computing phoneme-grapheme consistencies in accordance with Kessler and Treiman (2001) (Kessler & Treiman, 2001), Juul (2008a, 2008b) reported consistencies (on a scale from 0 to 1) of 0.672 for Danish vowels and 0.750 for consonants (Juul, 2008a). These coefficients imply that the correct spelling of a Danish phoneme is often relatively difficult to predict. Kessler and Treiman (2001) found an even lower vowel consistency of 0.529 for English (Kessler & Treiman, 2001).

Integrating movement in school teaching sessions has previously proven to be an effective strategy to support learning (Mavilidi et al., 2020). Therefore, it could potentially facilitate the attainment of reading skills. This approach can be implemented in many ways, for instance, by increasing children’s physical activity levels leading to the release of neurotransmitters that are beneficial to memory formation (Skriver et al., 2014). The majority of studies adopting this approach report a positive influence on academic performance, as shown in several reviews (Alvarez-Bueno et al., 2017; Daly-Smith et al., 2018; Donnelly et al., 2016; Norris et al., 2015; Singh et al., 2019).

Another way to apply movement in teaching is by embodied learning, where the movements support learning without a significant increase in the level of physical activity or metabolism. The theory of embodied cognition suggests that cognitive processes are mental representations derived from our senses (e.g., vision, hearing, kinesthetic, and tactile) and are integrated into the sensorimotor system (Lawrence W. Barsalou, 1999a, 1999b; Engelkamp & Zimmer, 1989; Glenberg, 2010; Macedonia, 2019). According to Barsalou, humans use their sensory neural structures to create multisensory representations of their environment and thereby are able to reuse those brain structures that are active during perception when mentally imagining an action or object (L. W. Barsalou, 1999a, 1999b; Lawrence W. Barsalou, 2008). Recruitment of the motor system seems to support cognitive processes when learning new complex tasks (Geary, 2008; Paas & Sweller, 2012). Thereby, bodily movement combined with academic learning will have the potential to enhance the ability to understand and provide better recall of the academic knowledge. Movements that are meaningfully integrated into the learning task have been recognized as especially beneficial (Skulmowski & Rey, 2018). Research has demonstrated that motor actions can enhance memory formation for specific information through gestures as well as observed enactment (Engelkamp & Zimmer, 1989; Madan & Singhal, 2012). Furthermore, observing gestures explaining or related to the learning task elicits stronger decoding than listening to the learning task alone (Engelkamp & Zimmer, 1989).

Based on this knowledge, educational research on embodied learning has investigated teaching models where the learning task is supported by, e.g., congruent bodily movements instead of only listening or observing in order to reinforce the learning process (Macedonia, 2019; Skulmowski & Rey, 2018). In fact, embodied learning does not necessarily involve bodily engagement of the learner’s own body; in addition to, e.g., gesturing and enactment, learning activities can also include watching animations or other seated interactions related to the content (Agostinho et al., 2015; Dubé & McEwen, 2015; Pouw et al., 2016). In a recent review by Skulmowski and Rey (2018), different types of embodied learning models were discussed with an educational focus, and a 2 × 2 grid taxonomy was designed with the following dimensions: task integration (incidental vs integrated) and bodily engagement (low vs high). According to Skulmowski and Rey, congruence between the learning task and bodily engagement is the primary key to enhanced performance, while the level of bodily engagement may have a lesser degree of influence (Skulmowski & Rey, 2018). A study by Wellsby and Pexman (2019) investigated whether the degree of sensorimotor experience modulates 5-year-old children’s word learning (Wellsby & Pexman, 2019). Results indicated that there was no effect in learning condition on children’s word recognition accuracy. The authors explain this by the lack of congruency between sensorimotor experience children received and the information to be learned. This empathizes the importance of congruency between learning task and embodiment. However, high bodily engagement has not only been linked to learning gains but also the risk of cognitive overload (e.g., Ruiter et al., 2015), and some researchers suggest thereby a medium degree of interactivity to be best suited for increasing learning outcome (Kalet et al., 2012).

Previous studies on children of 6–9 years old have shown that in both second language learning (Macedonia & Knösche, 2011; Mavilidi et al., 2015), mathematics (Beck et al., 2016; Goldin-Meadow et al., 1999), and letter recognition (Damsgaard et al., 2020), learning strategies integrating congruent bodily movements advanced academic performance (Macedonia, 2019). Mavilidi et al. (2015) demonstrated that children 4–5 years of age performing whole-body movements while learning new foreign words reached higher learning outcomes compared to children who used gestures or no bodily movements. This finding implies that whole-body movements may be more beneficial for word learning compared to part-body movements. However, in an acute study with 7-year-olds by Damsgaard and colleagues, a 10-min fine motor–enriched training (i.e., using hands and fingers) for distinguishing the letters b and d had a greater positive effect compared to gross motor-enriched training (i.e., using arms) and training without bodily movements (Damsgaard et al., 2020). Both studies suggest that the effect of integrated bodily movements may depend on the motor modality used. Other studies on the acquisition and development of reading and spelling skills have investigated bodily integration such as embodying letters (Botha & Africa, 2020) and walking the outline of a letter (Bara & Bonneton-Botté, 2017). The study by Botha and Africa (2020) investigated the effect of a perceptual-motor intervention for 6–7-year-old children delivered for 60 min twice a week over a period of 12 weeks. The study found that the perceptual-motor intervention was effective and reported a significant improvement in reading and spelling skills. Bara and Bonneton-Botté (2017) investigated the impact of a visuomotor intervention of 6 weeks, with six 45-min sessions for 5-year-olds’ cursive letter knowledge. They found a greater improvement in letter recognition following the visuomotor intervention, compared to a visual-only intervention.

Yet, none of these studies has compared whole-body movements and part-body movements with conventional non-motor teaching methods within the research area focusing on early reading development. Thereby, our intervention aims to clarify the effects of two embodied interventions for reading-related skills using movements with different motor modalities in close connection to the academic content. We designed a longitudinal study with an 8-week phonics intervention delivered three times a week for 30 min to investigate the effect of bodily engagement (whole-body and hand motor) on pre-reading and word reading skills. The outcomes of the interventions were compared to conventional, non-motor teaching normally implemented in schools.

The study addresses the following three questions:

1. *Does teaching condition (whole-body, hand motor, and non-motor teaching) result in significant group differences in letter-sound knowledge and reading of trained words?*
  More specifically, we investigated if teaching with different degrees of motor modality would affect children’s knowledge of letter-sound connections trained in the intervention (divided into standard and conditional sounds) and reading trained words immediately post-intervention and at 17–22 weeks after the intervention (retention test). We tested the hypothesis that children in the intervention groups connecting hand motor or whole-body movements and letter-sounds would have the greatest direct training effect in letter-sound connections (standard and conditional sounds) and word reading.
2. *Does teaching condition (whole-body, hand motor, and non-motor teaching) result in significant group differences in word reading?*
  Specifically, we tested if the interventions would affect children’s ability to read words in a standardized test (far transfer effect) and their ability to read short words that resembled the words trained during the intervention (near transfer effect). We tested the hypothesis that children in the intervention groups connecting hand motor or whole-body movements and letter-sounds would have the greatest transfer effect in word reading.
3. *Is there a significant correlation between children’s baseline motor skills and children’s reading-related skills post-intervention and at retention test?*

We aimed to clarify the role of motor skills in relation to reading-related skills. We employed two motor skill measures and tested the hypothesis that motor skill ability was associated with children’s pre-reading and word reading skills. Furthermore, we asked whether children’s motor skills could be associated with the prevalence of performing embodied learning exercises when producing letter-sounds.

## Materials and Methods

### Study Design and Participants

The present study was conducted with 5–6-year-old children who had just started school (grade 0) and were recruited from 10 different classes from four elementary schools in the Copenhagen area, Denmark. At the beginning of grade 0, students are usually able to identify at least half of the letters of the alphabet (Juul, 2008b), but only few students will be able to read words (Juul, 2008b; Poulsen & Jensen, 2015). One hundred eighty-three children were included in the study after obtaining written consent from parents. Fourteen children were excluded due to less than 90% presence of total lessons from the intervention, eight children with neural developmental conditions were excluded, and one child withdrew from the study, which in the end resulted in 149 participating children (76 girls, 73 boys, mean age ± SD = 6.2 ± 0.4) (see Table 2 for demographic characteristics by intervention group).

The study was approved by the local Ethical Committee at University of Copenhagen, Denmark (protocol: 504–0032/18–5000), registered in ClinicalTrials.gov (NCT04618822), and carried out in accordance with the Helsinki Declaration II. The present study is described in detail elsewher﻿e (Gejl et al., 2021), and only methods pertinent to this study are included here.

### Intervention

The study is a three-armed randomized controlled trial including two intervention groups and one control group. One hundred eighty-three participating children were individually randomly assigned before baseline assessment to receive either teaching activities with whole-body movements (WM), hand movements (HM), or a control group with no movements besides handwriting (CON) over an 8-week period. Within each class, six participants were allocated to WH and six participants to HM, and the remaining participants were constituted to CON in an approximate 1:1:2 fashion. The intervention sessions were delivered during the same time slot but in separate classrooms for each group. In addition, the control group learning activities were incorporated into the school curriculum for the period of the intervention. This meant that all children in the participating classes completed the control group activities, but only the children whose parents provided written consent were included in the control group.

During the 8-week intervention period, three sessions of 30-min duration were completed each week, counting 24 sessions in total. Three classes were delayed 1 week, and one of the three classes only performed 23 of 24 sessions due to COVID-19. The learning activities focused on the acquisition of letterforms, letter-sound correspondences, and reading and spelling short words. Thus, word meaning was not a primary focus in this intervention. In all three groups, the learning content of the activities was identical. However, the groups varied with regard to the degree of bodily movement. The first 6 weeks followed the same weekly structure involving the same type of activities, and every week, four or five new letters and related sounds and two to four new words (target words) containing the letters and sounds were presented for the children. The last 2 weeks consisted of the repetition of the first 6 weeks, with a specific focus on letters with more than one pronunciation. Thereby, the children were taught 25 letters in total and their related sounds (standard and conditional pronunciations) and 18 target words during the intervention period. The intervention material was developed based on the research-founded Danish teaching material, Fandango Mini, which is recognized and used by several preschool teachers in Denmark (Jacobsen & Veber Nielsen, 2011). Fandango Mini is based on a synthetic phonics approach and is scheduled as a 20-week systematic course covering both standard and conditional pronunciations of the letters. From the very beginning, students practice reading and spelling of words composed of the letter-sounds trained so far (Gejl et al., 2021).

Teachers and instructors were asked to keep a written log of attendance for each child. Moreover, teachers and instructors were asked to note how many exercises within each session were completed and to what degree it followed the protocol. If any deviations from the protocol were observed, oral guidance was given to the instructor/teacher by the research team.

### Intervention Conditions

The two interventions groups (HM/WM) varied in regard to the motor modality (hand vs. whole-body movements). The participating children were taught to make specific movements to letter-sounds (phoneme movements), and these movement-sound couplings were used throughout the intervention. The phoneme movements were executed from left to right, following the reading direction, and the movements were also associated with objects or living creatures (e.g., the movement coupled to the sound “S” was associated with a snake). Long letter-sounds (e.g., the movement coupled to the letters “O,” “A,” “S”) were performed as slow and fluent movements, while staccato letter-sounds were carried out as fast and powerful movements (e.g., “K,” “T,” “P”).

Children in the hand movement (HM) group performed the movements only using their arms and hands seated on a chair around a table with one trained instructor. The activities were performed individually or in randomly allocated pairs.

The participants in the whole-body movement (WM) group were standing in a circle on the floor with one trained instructor, and they were encouraged to use their whole bodies to make the phoneme movements individually or in random allocated pairs. For both groups, it was important that the children performed the respective phoneme movements while pronouncing the letter-sound so there was a strong movement-sound coupling.

### Control Condition

Children in the control condition (CON) performed activities similar to the two intervention groups with a strong focus on letter-sounds. However, the children in the control condition did not perform movements beside handwriting. The activities were performed seated on a chair, individually or in random allocated pairs, using pencil and paper and were administrated by their own teacher. Therefore, the control condition served to follow a protocol that is typically delivered in schools, though closely matched to the intervention groups. The amount of time the intervention group practiced phoneme movements, the intervention group worked on letter-sound coupling. We recognize the nature of embodied learning theory and that our control group still performs embodied learning as the group uses handwriting and various senses (e.g., hearing) used traditionally in teaching situations. From now on, this group will be described as being non-embodied since our two intervention groups perform embodied learning to a larger degree using whole-body and hand motor movements.

**Fig. 1:**
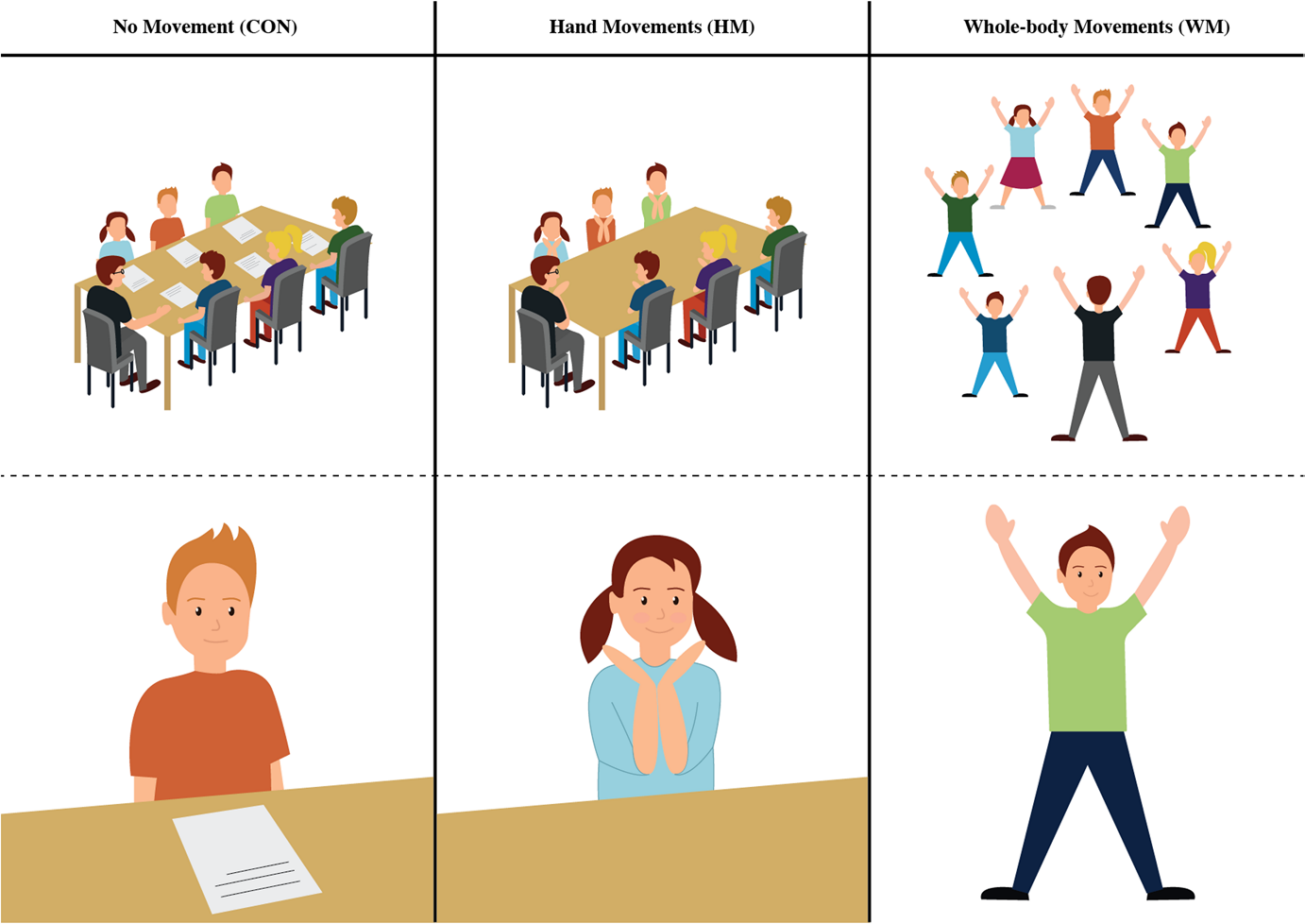
Condition overview. Overview of conditions and movement/no movement in control group (CON), hand movement group (HM), and whole-body movement (WM) group. The HM group only performed movements using arms and hands. The WM group used their whole body to form the shapes of the phonemes. Illustrated is the children performing the phoneme of the letter “Y”

Figure 1 illustrates the conditions in CON, HM, and WM groups.

### Test Procedures

**Fig. 2:**
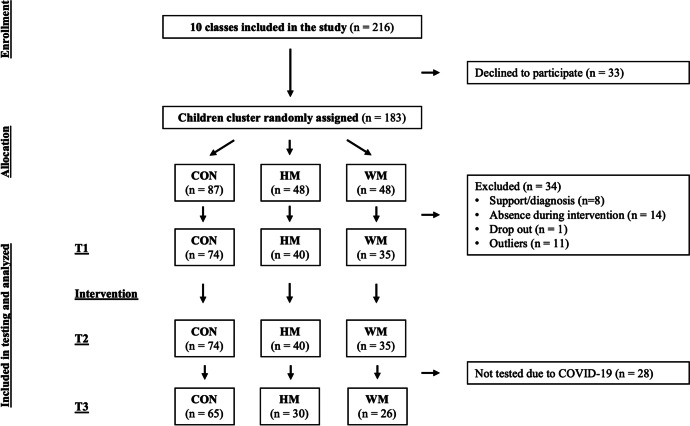
Flow diagram of this study. Two hundred sixteen children were invited to the project (10 classes from four different schools). Thirty-three children declined to participate. One hundred eighty-three children were randomly assigned to either CON (no-movement group), HM group (making hand phonemes using only arms and hands), or WM (making body phonemes using their whole body). Thirty-four children got excluded due to diagnosis, absence during intervention, dropout, and outliers. The analysis is based on 74 children from CON, 40 from HM, and 35 from WM. Due to COVID-19, one school was closed which resulted in 28 children not tested at the retention test

Age, sex, handedness, bilingualism, height, and weight were collected prior to baseline measures (T1). To evaluate the effect of the interventions, all measures of reading-related skills and motor skills were administrated at the schools by trained instructors before (T1), after (T2) the 8-week intervention period, and after a retention period of 17–22 weeks (T3) though the original protocol planned for the retention test to take place after 8 weeks. This was not possible due to COVID-19 (see flowchart Fig. 2.)

Six of the tests assessing reading-related skills and motor skills were conducted individually (IT) with a trained instructor in a one-to-one session. Three tests were conducted in groups (GT) of 12 participants separated from one another to avoid copying. The group tests were delivered by two trained instructors. The individual tests and the group tests were performed on two separate days, and the test duration for each child was approximately 60 min in total. All data were two-factor registered by two instructors. Discrepancies were resolved by further review of the data by authors who did not register the data in the first place (LD, SKJ).

### Measures

#### Standardized Test

Two tests were performed to evaluate the children’s word reading and letter-sound identification.

1. **Standardized Word Reading Task (GT)**
  The test evaluated children’s word reading accuracy and efficiency. This test is widely used in the Danish school system and was administrated strictly according to the manufacturer’s description (Juul & Møller, 2010). The test consisted of 78 items (preceded by two practice items). For each item, the children had to select one of four pictures that corresponded to a printed word. The child should solve as many items at possible within a time limit of 4 min. After every minute, the child was asked to change the pencil color in order to monitor their progression throughout the test. The test outcomes included the number of correctly solved items (efficiency) and the percentage of correctly solved items (accuracy) used as a measure of the transfer effect.
2. **Standardized Letter-Sound Identification Task (GT)**

Similar to the Word Reading Test, this test is also widely used in the Danish school system. The test evaluated children’s ability to identify the first letter in a word read aloud. The test was administrated strictly according to the manufacturer’s description (Møller & Juul, 2013) and contained 20 items (preceded by two practice items). Each item consisted of a picture followed by five letters. The words represented by the picture were read aloud by the instructor, and the participants had to mark the first letter of each word. The test outcome was the total number of correct items (out of 20 possible points) and used as a baseline measure of children’s letter-sound knowledge.

#### Word Reading

Two tests assessed children’s word reading.

1. **Word Reading with Pictures (IT)**
  To evaluate children’s ability to read the words they had been presented with during the intervention (target words), we used a computer-based test made specifically for this intervention. The test consisted of 18 representations of target words, one word per presentation. A detailed list of target words and pronunciations can be found in Table 1. Below the presented target word, four pictures were placed where only one illustrated the target word from the intervention. The remaining three pictures represented words (distractors) that had either the initial (two pictures) or the final (one picture) sound in common with the target word. To give the correct answer, participants had to select the target word picture on the touch screen. They were asked to touch the correct picture as fast as possible. The presentations of the target word and the order of the four pictures were randomized between participants and time points. The internal consistency of the test evaluated by the Kuder-Richardson formula 20 has previously been analyzed to 0.872 (95% CI 0.866–0.879) (Malling et al., 2021). Word Reading with Pictures has previously shown to correlate significantly with the standardized word reading task accuracy (Malling et al., 2021). The outcomes from the test constituted the mean response time for correct answers and the number of correct answers and were used as a measure of the direct training effect.

**Table 1:** Letters, letter-sounds, and target words trained during each week of the intervention period

Week	1							2					3						4					5					6					7 and 8
**Letters**	l	å	s	m	e			b	i	u		y	n	k	f	a		æ	h	t	d		g	p	o	j		ø	r		c	v		all
**Letter-sounds**	[l]	[ɔ]	[s]	[m]	[e]	[ɛ]	[ə]	[b]	[i]	[u]	[ɔ]	[y]	[n]	[k]	[f]	[a]	[ɑ]	[ɛ]	[h]	[t]	[d]	[ð]	[g]	[p]	[o]	[ʌ]	[j]	[ø]	[ʁ]	[ɐ̯]	[s]	[v]	[w]	all
**Target words in Danish [pronunciations]**	lås [ˈlɔˀs]							bus [ˈbus]					fly [ˈflyˀ]						dæk [ˈdεg]					gås [ˈgɔˀs]					sø [ˈsøˀ]					båd [ˈbɔˀð]
	mel [ˈmeˀl]							bil [ˈbiˀl]					nul [ˈnɔl]						hat [ˈhad]					kop [ˈkʌb]					rive [ˈʁiːvə]					mur [ˈmuɐ̯ˀ]
																			hest [ˈhεsd]					majs [ˈmɑjˀs]					hav [ˈhɑw]					
																								kano [ˈkæːno]										
**Translated target words to English**	lock							bus					airplane						rire					goose					lake					boat
	flour							car					zero						hat					cup					rake					wall
																			horse					corn					sea					
																								canoe										

Eighteen Danish target words were trained in the intervention. A translation has been performed from Danish to English for further understanding for the reader of this paper
2. **Word Reading without Pictures (IT)**

To test the potential transfer effects of the intervention on children’s ability to read short words that resembled the target words from the intervention, a computer-based test made specifically for this intervention was performed. The test consisted of 12 untrained words (no targets words). The words were presented one at a time with different letter lengths (four 2-letter words, four 3-letter words, and four 4-letter words) for up to 16 s or until an answer was given. Two versions of the test were delivered: one conducted at baseline (T1) and one conducted post-intervention (T2). At the retention test (T3), the test delivered at baseline was completed again. All versions of the test and the selection of words with the same number of letters were presented randomly. The child was instructed to read the word and say it aloud. The assessor registered the answers as correct or incorrect by pressing a green or red button on the keyboard, respectively. If no answer was given within the time limit, a new word appeared on the screen. If incorrect or no answers were provided for all four 2-letter words, the test ended. The internal consistency of the two test versions evaluated by the Kuder-Richardson formula 20 has previously been analyzed to 0.896 (95% CI 0.887–0.905) and 0.891 (95% CI 0.881–0.900) (Malling et al., 2021) for versions 1 and 2, respectively. Word Reading without Pictures has also shown to correlate significantly with standardized word reading task accuracy (Malling et al., 2021). The test outcome was the number of correctly read words, and it was used as a measure of the transfer effect.

#### Letter Knowledge

Three tests were performed to evaluate children’s letter knowledge.

1. **Letter Naming (IT)**
  A Danish version of the DIBELS Letter Naming Fluency test was used which consisted of 12 rows of 10 letters (mixed upper and lower case) on a piece of paper (A4) (Good & Kaminski, 2002; Poulsen & Jensen, 2015). The child was asked to name as many letters as possible in 1 minute while pointing at each letter. Wrong letter-names were registered by the instructor. If the child did not name a letter within 3 seconds, the instructor said the letter name and encouraged the child to name the next letter. Prior to commencing the test, the child was provided with a row of 10 letters as practice. The total number of all correctly named letters was used as a baseline measure of children’s letter knowledge.
2. **Naming of Letter-Sounds (Incl. the Use of Movement) (IT)**
  To assess children’s knowledge of letter-sounds, they were asked to pronounce the sounds of letters “a,” “d,” “e,” “o,” “r,” “u,” and “v” which have several possible pronunciations in Danish. In total, the test assessed the knowledge of seven standard letter-sounds and eight conditional letter-sounds. The instructor read aloud the letter-names one at a time. The child was standing up while answering and thereby had the opportunity to make movements to the sounds. For every letter-sound, the child’s answer was registered as correct/incorrect/missing, and it was recorded whether any movement was used. The result of the test was the number of correct letter-sounds pronounced (1) in total, (2) as standard letter-sounds, and (3) as conditional letter-sounds. The test was used as a measure of the direct training effect. The internal consistency of the test evaluated by the Kuder-Richardson formula 20 has previously been analyzed to 0.73 (95% CI 0.72–0.75) (Malling et al., 2021). The number of correct letter-sounds names in total has shown to correlate significantly with the Standardized Letter-Sound Identification Task (Malling et al., 2021).
3. **Letter-Sound Matching (GT)**

To evaluate children’s knowledge of letter-sound correspondences trained during the intervention, a simple multiple-choice test was constructed on paper. The test consisted of 15 trials. In each trial, a sound corresponding to a standard or a conditional pronunciation of a letter was given by the instructor. Children were instructed to identify the sound and match it to the correct letter given a choice of four letters presented in a row on the paper (preceded by one practice trial). The internal consistency of the test have been evaluated by the Kuder-Richardson formula 20 to 0.67 (Malling et al., 2021). Letter-Sound Matching has also shown to correlate significantly with the Standardized Letter-Sound Identification Task (Malling et al., 2021). The 15 sounds represented both standard and conditional pronunciations of the letters “a,” “e,” “o,” “r,” “u,” and “v.” The test outcomes included the number of correct letter-sound matches (1) in total, (2) as standard letter-sounds, and (3) as conditional letter-sounds. The test was used as a measure of the direct training effect.

### Motor Skills

Two tests were performed to evaluate children’s fine and gross motor skills.

1. **Flamingo Balance Test (IT)**
  The flamingo balance test is a standardized test to assess the gross motor skill of balancing on one leg (Adam, Klissouras, Ravazzolo, & Renson, 1987). Children were asked to stand for 1 min with one leg on the floor and the other leg bent backwards. The hand on the same side as the bent leg grasped the food. To get familiar with the test, children had one trial before the actual test. The number of attempts needed to stand on one leg for 1 minute was recorded for each leg. Children who needed more than 15 attempts within the first 30 seconds were excluded from this test. The test was only performed at T3. The test outcome was the sum of attempts with both legs where lower scores indicate better performance. The test was used to compute a total motor skill score together with the outcomes of the 9-Hole Pegboard Test described below.
2. **9-Hole Pegboard Test (IT)**

The 9-hole pegboard is a standardized test that has previously been used to evaluate children’s fine motor skills (Longcamp et al., 2005; Smith et al., 2000). The test consisted of a board with nine 1.3 cm (0.5 in) deep holes that were spaced 3.32 cm (1.25 in) apart. Sitting on a chair with the board placed in front of them, children were instructed to pick up the pegs one at a time and to put them into the holes in any order until all of the holes were filled. Then, they were asked to remove all of the pegs in any order, one at a time. There was one untimed practice trial followed by the test, which was timed with a stopwatch starting when the child touched the first peg until the last peg was removed. The test was conducted twice, once with the dominant hand and once with the opposite hand. The test outcomes included dominant and opposite hand completion times. The test was used to perform a total motor skill score together with the outcome of the flamingo balance test.

### Statistical Analyses

Statistical analyses were performed in R studio (RStudio, 2020).

Each baseline measure was compared between the intervention groups and control group using a one-way analysis of variance (ANOVA), and chi-square tests were used for the categorical measures (bilingual, dominant hand, and sex). Children with test results of more than ± 2SD from the mean in two or more tests were considered outliers and were excluded from all analyses (*n* = 11). In total, data were analyzed based on 74 children in CON, 40 children in HM, and 35 children in WM (149 children in total).

Data from letter knowledge, word reading, standardized test, and motor skills performance were analyzed using linear mixed models with group-time interactions as fixed effects, using R package lme4 (Bates, Mächler, Bolker, & Walker, 2015). The choice of using linear mixed models was especially beneficial as they allow to account for missing data (e.g., absent at test day). The data was analyzed using group-time interaction effect with CON, HM, and WM as groups and time as measures taken at T1, T2, and T3. “Subjects” and “school” were added to the model as random effects and “age” as fixed effect since children’s letter knowledge are age-dependent. Pairwise comparisons between delta values were used to characterize interaction effects. To reduce the problem of multiple testing, only relevant model-based specified comparisons were performed using the *emmeans* R package (Searle, Speed, & Milliken, 2021). *p* value adjustment was based upon the Tukey method for comparing a family of three estimates. The level of statistical significance was set to *p* < 0.05.

Cohen’s *d* effect sizes (Cohen, 2013) were calculated as the mean differences in performance divided by the pooled standard deviations. Influenced by Cohen’s convention regarding magnitude of effect sizes, a Cohen’s *d* effect size in the range 0.2–0.35 was considered small, in the range 0.35–0.65 moderate and > 0.65 large (Cohen, 2013). 

Correlational analysis were performed using Spearman’s rank correlation (Savicky, 2015). This test is nonparametric and more robust for small sample sizes. Spearman’s rank correlation was used to study the association between motor skill performance and letter-sound knowledge.

## Results

### Baseline Characteristics

The chi-square tests and one-way-ANOVAs revealed no significant between-group differences for demographic data at T1 (*p* > 0.05) (Table 2) nor for the baseline variables on word reading and letter knowledge (*p* > 0.05) (Table 3).

**Table 2:** Demographics of the three groups (CON, HM, WM)

	CON	HM	WM
Participants (n)	74	40	35
Age (years)	6.2 ± 0.4	6.3 ± 0.4	6.3 ± 0.3
Height (cm)	122.8 ± 4.7	122.0 ± 4.8	122.1 ± 5.6
Weight (kg)	23.2 ± 3.2	22.8 ± 3.8	22.8 ± 3.0
BMI (kg/m^2^)	15.3 ± 1.7	15.2 ± 1.7	15.2 ± 1.2
Sex (% girls)	55.41	52.50	40.00
Bilingualism (% bilingual)	25.68	25.00	14.29
Dominant hand (% right)	93.24	92.50	88.57

Data reported as mean ± SD. No significant between-group differences were observed for any of the measures. CON, control group; HM, hand movement group; WM, whole-body movement group

**Table 3:** Performance at T1 (baseline) for the three intervention groups. Data reported as mean ± SD

Measure	CON	HM	WM
Standardized test			
Standardized letter identification task (no. of correct answers, max = 20)	8.5 ± 4.6	9.5 ± 5.2	8.1 ± 3.8
Standardized word reading (no. of correct answers in 4 min)	3.5 ± 2.4	3.6 ± 2.7	3.0 ± 2.0
Word reading			
Word reading with pictures (% of correct answers)	27.6 ± 11.5	27.0 ± 10.9	28.4 ± 13.9
Word reading with pictures (reaction time, s)	8.2 ± 5.5	8.3 ± 6.0	8.1 ± 6.5
Word reading without pictures (no. of correct answers, max = 12)	0.1 ± 0.6	0.5 ± 1.2	0.1 ± 0.4
Letter knowledge			
Naming of letter-sounds (no. of correct answers, max = 15)	3.5 ± 2.3	4.2 ± 2.5	3.0 ± 2.5
Naming of letter-sounds (no. of correct answers, max = 7): standard	3.3 ± 2.2	3.9 ± 2.3	2.9 ± 2.3
Naming of letter-sounds (no. of correct answers, max = 8): conditional	0.2 ± 0.4	0.3 ± 0.5	0.1 ± 0.3
Naming of letter-sounds, movement (% of correct answers)	8.5 ± 25.6	5.9 ± 16.4	3.8 ± 19.6
Naming of letter-sounds, movement (% of correct answers): standard	8.8 ± 26.5	6.4 ± 17.6	3.8 ± 19.6
Naming of letter-sounds, movement (% of correct answers): conditional	9.1 ± 30.2	0.0 ± 0.0	0.0 ± 0.0
Letter-sound matching (no. of correct answers, max = 15)	6.3 ± 2.5	6.5 ± 2.5	6.0 ± 2.6
Letter-sound matching (no. of correct answers, max = 7): standard	3.9 ± 1.7	4.1 ± 1.9	3.8 ± 1.9
Letter-sound matching (no. of correct answers, max = 8): conditional	2.5 ± 1.5	2.3 ± 1.2	2.2 ± 1.6
Letter naming (no. of correct answers)	12.5 ± 10.3	14.1 ± 14.5	14.2 ± 10.0
Motor skills			
Flamingo Balance (total touch downs left/right)	8.0 ± 6.9	5.2 ± 5.1	8.4 ± 8.8
9-Hole Pegboard (total time spent (s) dominant/nondominant)	58.6 ± 9.4	56.3 ± 8.6	57.3 ± 8.1

The standardized measures suggested that the sample studied was fairly typical of Danish grade 0 with respect to letter-sound identification with a mean score of 8.68, which is slightly under the published norm of 10.0 (Møller & Juul, 2013). For standardized measures of word reading, the present study’s sample mean score of 3.4 was somewhat lower than the earlier published norm from a study with children at the end of grade 0 (17.8) (Malling et al., 2021). The same was seen for letter naming fluency with a mean score of 13.37 compared to published norm from Malling et al. (2021) of 38.49. The baseline characteristics for the sample studied are thereby expected since the participating children started attending grade 0 only 1 month prior to testing.

To investigate whether the teaching condition (CON, HM, WM) had a direct training effect on children’s letter knowledge and word reading skills post-intervention and at retention (research question 1), analysis was performed on the following three measures: Naming of Letter-Sounds, Letter-Sound Matching, and Reading Test with Pictures.

**Fig. 3:**
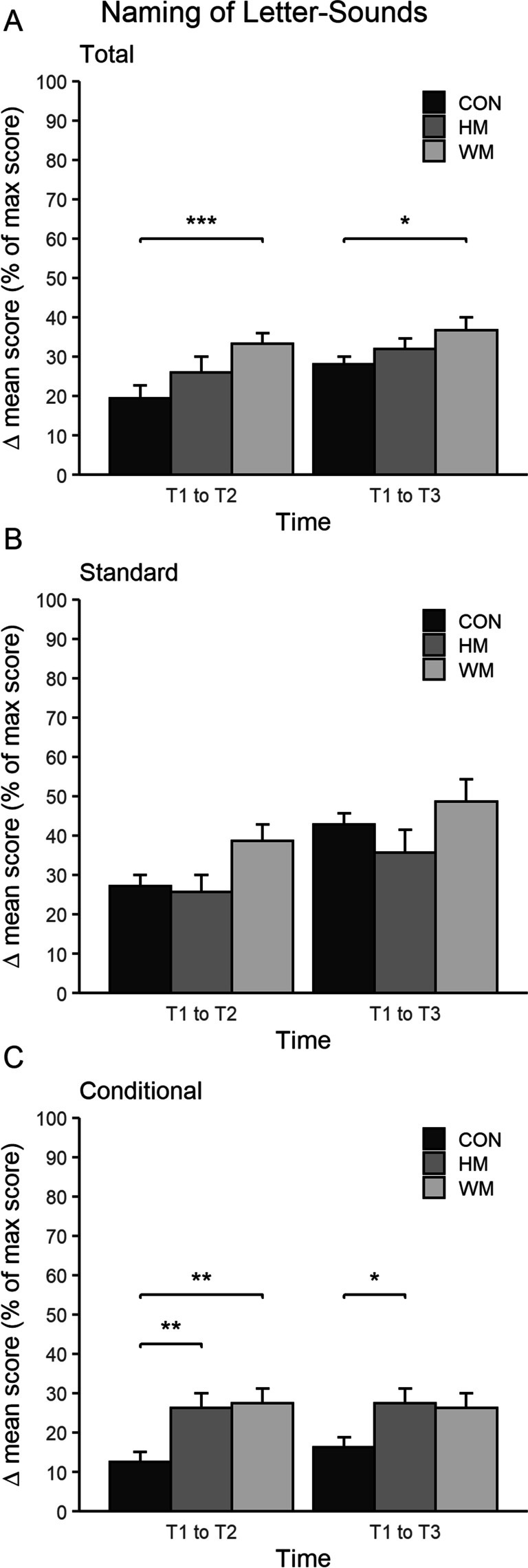
Results of children’s ability to name letter-sounds for a given letter*.*** A** The mean delta score of % max score (*y*-axis) of naming letter-sounds (both standard and conditional pronunciations) within the groups CON, HM, and WM. A significant difference between WM and CON was seen from T1 to T2 (***) and T1 to T3 (*). **B** The mean delta score of % max score (*y*-axis) of naming *standard* letter-sounds within the groups CON, HM, and WM. No significant differences between the groups were seen.** C** The mean delta score of % max score (*y*-axis) of naming *conditional* letter-sounds within the groups CON, HM, and WM. A significant difference was seen from T1 to T2 (**) for both WM and HM compared to CON. From T1 to T3, a significant difference (*) was seen between HM and CON. *, *p* < 0.05; **, *p* < 0.01; ***, *p* < 0.001

*Naming of Letter-Sounds*: A between-group analysis showed that WM improved their ability to name letter-sounds correctly significantly more than CON both from T1 to T2 (CON^T1–T2^ = 82% versus WM ^T1–T2^ = 167%, *p* < 0.001, *d* = 2.8) and from T1 to T3 (CON^T1–T3^ = 120% versus WM ^T1–T3^ = 183% *p* < 0.05, *d* = 0.7) (see Table 4 for absolute values and Fig. 3 for percentages). Further, it was found that both the hand motor movement group (HM) and the whole-body movement group (WM) improved significantly better at naming conditional letter-sounds compared to the control group (CON) from T1 to T2 (WM^T1–T2^ = 1050% and HM^T1–T2^ = 700% versus CON^T1–T2^ = 500%, *p* < 0.01, *d* = 0.6, *p* < 0.01, *d* = 0.5) and also from T1 to T3 for the HM group compared to CON (CON^T1–T3^ = 650% versus HM ^T1–T3^ = 733% (*p* < 0.05,* d* = 0.5).

**Table 4:** Improvement from pre (T1) to post (T2) or retention test (T3)

Measure (delta)	T1 > > T2			T1 > > T3		
	CON	HM	WM	CON	HM	WM
Standardized test						
Standardized word reading (no. of correct answers in 4 min)	5.3 ± 1.1	5.6 ± 1.4	6.7 ± 1.5	13.1 ± 1.1	15.1 ± 1.6	16.8 ± 1.7
Word reading						
Word reading test with pictures (% of correct answers)	20.0 ± 3.2	20.7 ± 4.2	13.5 ± 4.5	30.9 ± 3.3	39.4 ± 4.7	32.7 ± 5.0
Word reading test with pictures (Reaction time, s)	0.1 ± 0.8	− 0.1 ± 1.1	0.5 ± 1.2	− 0.3 ± 0.8	− 0.7 ± 1.2	− 0.3 ± 1.3
Word reading test without pictures (no. of correct answers, max = 12)	1.9 ± 0.4	2.0 ± 0.6	2.1 ± 0.6	4.8 ± 0.5	5.3 ± 0.7	4.7 ± 0.7
Letter knowledge						
Naming of letter-sounds (no. of correct answers, max = 15)	2.9 ± 0.3	3.9 ± 0.4	5.0 ± 0.4^a^	4.2 ± 0.3	4.8 ± 0.4	5.5 ± 0.5^a^
Naming of letter-sounds (no. of correct answers, max = 7): standard	1.9 ± 0.2	1.8 ± 0.3	2.7 ± 0.3	3.0 ± 0.2	2.5 ± 0.4	3.4 ± 0.4
Naming of letter-sounds (no. of correct answers, max = 8): conditional	1.0 ± 0.2	2.1 ± 0.3^a^	2.2 ± 0.3^a^	1.3 ± 0.2	2.2 ± 0.3^a^	2.1 ± 0.3
Naming of letter-sounds, movement (% of correct answers)	0.1 ± 4.5	64.8 ± 5.9^a^	66.5 ± 6.6^a^	− 2.8 ± 4.6	15.8 ± 6.5	30.3 ± 7.2^a^
Naming of letter-sounds, movement (% of correct answers): standard	− 0.5 ± 4.6	62.9 ± 6.0^a^	63.8 ± 6.6^a^	− 4.4 ± 4.7	14.4 ± 6.7	28.5 ± 7.2^a^
Naming of letter-sounds, movement (% of correct answers): conditional	7.2 ± 10.9	91.9 ± 10.8^a^	73.0 ± 16.2^a^	5.3 ± 10.7	27.5 ± 11.6	37.6 ± 16.3
Letter-sound matching (no. of correct answers, max = 15)	3.1 ± 0.3	4.3 ± 0.4	3.7 ± 0.5	4.2 ± 0.3	4.9 ± 0.5	5.2 ± 0.5
Letter-sound matching (no. of correct answers, max = 7): standard	1.8 ± 0.2	2.1 ± 0.3	2.0 ± 0.3	2.4 ± 0.2	2.2 ± 0.3	2.3 ± 0.4
Letter-sound matching (no. of correct answers, max = 8): conditional	1.2 ± 0.3	2.1 ± 0.3	1.7 ± 0.4	1.7 ± 0.3	2.6 ± 0.4	2.9 ± 0.4^a^

Data reported as estimated mean ± SE. ^a^, significant different from CON

**Fig. 4:**
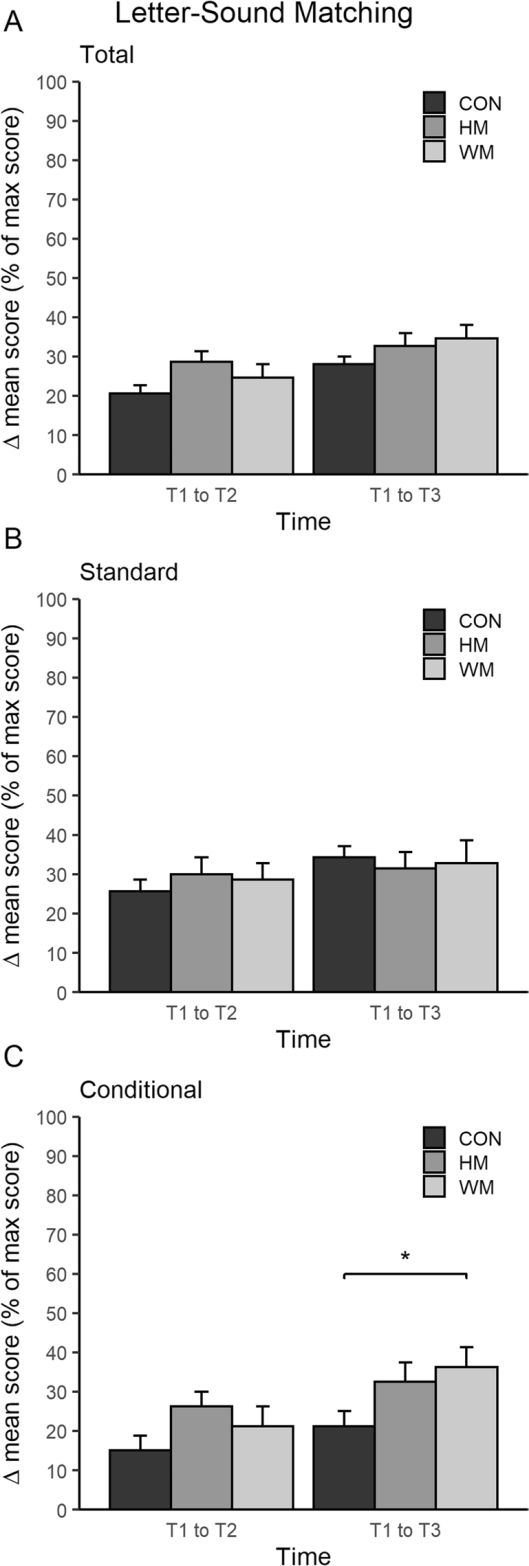
Results of children’s ability to match letters with letter-sounds. **A** The mean delta score of % max score (*y*-axis) of matching sounds to a letter (both standard and conditional letter-sounds) within the groups CON, HM, and WM. No significant differences between groups were seen from T1 to T2 and T1 to T3. **B** The mean delta score of % max score (*y*-axis) of matching standard letter-sounds to a letter within the groups CON, HM, and WM. No significant difference between groups was seen from T1 to T2 and T1 to T3. **C** The mean delta score of % max score (*y*-axis) of matching conditional letter-sounds to a letter within the groups CON, HM, and WM. A significant difference (*) was seen from T1 to T3 between WM and CON. *, *p* < 0.05; **, *p* < 0.01; ***, *p* < 0.001

*Letter-Sound Matching*: There were no between-group differences on the total score or standard letter-sounds, but for the conditional letter-sounds, there was a significant difference between WM and CON from T1 to T3 (CON^T1–T3^ = 68% versus WM ^T1–T3^ = 132%, *p* < 0.05,* d* = 3.0; see Table 4 for absolute values and Fig. 4 for percentages).

No significant differences were seen in Reading Test with Pictures.

Following both WM and HM interventions, it was evident that movements became an important part of children’s processing of letter-sounds. During the letter-sound test, none of the children in the CON group supported their answers with movements. In contrast, pooled data from WM and HM groups showed that 53% of children supported their answers with movements. Due to the significant difference between the groups in the naming of letter-sounds, we wanted to investigate if children’s motor skill performance would be associated with their choice to use movement while pronouncing the letter-sound (research question 3). An exploratory Spearman correlation showed no significant correlation (*p* > 0.05) between the children’s motor skills performance score and the prevalence of using movement while naming letter-sounds. This might indicate that there is no relationship between children’s degree of motor skill performance score and their ability to learn letter-sound correspondences by embodied learning.

## Discussion

### Embodied Learning Effect on Children’s Pre-Reading Skills

The present study provides supportive evidence to our hypothesis that embodied learning activities have an effect on children’s letter-sound knowledge learning. We found that using whole-body movements and hand motor movements while learning letter-sound correspondences was associated with better learning outcomes compared with non-embodied training post-intervention. When looking more deeply into the types of letter-sounds, it was found that children from both intervention groups (whole-body and hand motor) improved in their ability to recall conditional letter-sounds post-intervention. The fact that the intervention effect was mainly seen on the conditional letter-sounds should probably be explained by the order in which children usually acquire the sounds of letters. Initially, children acquire the standard sounds of the letters, which to a large extent correspond to the names of the letters (Treiman et al., 1998). At the beginning of grade 0, students already know at least half of the letters of the alphabet (Juul, 2008b), and when children know the names of the letters, they are not far from knowing their standard sounds as well. Typically, children need to master the standard sounds of the letters before acquiring their conditional sounds (Elbro, 2013). Children can either learn the conditional sounds directly through teaching or indirectly by mapping the spelling of words with their pronunciation while decoding them. Since the intervention took place at the beginning of grade 0, most children had no prior knowledge of conditional letter-sounds, and so, they needed to learn and recognize these sounds as *alternative* pronunciations of the letters alongside their names and standard pronunciations. Altogether, these results suggest that learning of letter-sound correspondences with the integration of congruent bodily movements may have an advantage over conventional methods.

When comparing present results with children who attended school for 4 more months following standard curriculum for grade 0 (Malling et al., 2021), we see that the hand motor movement group and whole-body movement group perform significantly better when recalling conditional letter-sounds (~ 15% vs 7% correct answers) at T2 (data not shown). We found no evidence to suggest that letter-sound knowledge differed in the WM group when tested immediately after the intervention and following the retention period. This indicates a high likelihood of maintaining the acquired knowledge. Engelkamp and Zimmer (1989) argued that information is memorized better if the learner performs the described action during learning compared to just getting verbal information, also known as the enactment effect. According to this theory, the embodied learning groups should memorize the letter-sounds and letter-sound movements better compared to the children who participated in conventional verbal learning. This emphasizes the powerful effect of embodied learning on children’s pre-reading skills.

These findings are in accordance with previous studies that have shown the association between the integration of bodily movements during academic learning (e.g., new foreign words) and higher learning outcomes (Mavilidi et al., 2015). The interventions delivered in the current study had the unique merit of integrating meaningful movements that were closely associated with the corresponding letter-sounds, and it suggests that the delivered exercises had a sufficient cognitive load level without risk of cognitive overload (Sweller, 2011). As seen in previous research on enactment and gesturing, learning involving meaningful bodily movements enhances memory for specific information (e.g., letter-names and letter-sounds) caused by more elaborate representations following motor engagement (action-event memory formation), which may explain the present study results (Engelkamp & Zimmer, 1989; Madan & Singhal, 2012).

### Transfer Effects to Word Reading Skills

Considering the significant improvement in letter-sound knowledge in both movement groups, we expected that the interventions would also facilitate the transferability of the learnt skills. However, no transfer effects were observed, and our hypothesis was not supported. Several reasons might explain this finding.

Before children can understand the alphabetic code, that sounds and letters can combine, which, in turn, will allow for the development of basic word reading skills, they need both phoneme awareness and letter knowledge (e.g., Bowey, 2005). So, to observe a transfer effect from T1 to T2 on word reading, the children participating in the 8-week intervention would have had to acquire letter-sound knowledge proficiency *and* translate their newly gained knowledge into a basic word reading strategy. Our data show that this was not obtained. If we use an accuracy level of 70% correct for reading short regular words (based on word reading without pictures) as a criterion for the achievement of basic word reading skills as suggested by Juul et al. (2014), we find that only 4% of all children acquired basic word reading skill during the intervention period. Further, at T3, 7–8 months after school starts, the proportion of children that had acquired basic word reading skills had risen to 29%. This corresponds to the proportion found in a study by Malling et al. (2021) after 7 months of semiformal literacy instruction common of Danish schools (Malling et al., 2021). Hence, regardless of teaching condition, the intervention did not boost children’s development of basic word reading skills.

We have no specification of the literacy instruction the children took part in during the retention period, but it might be the case that the children did not have had sufficiently word reading practice neither during the intervention period nor during the retention period to profit from their enhanced letter-sound knowledge. While some intervention activities involved the combination of spelling and reading to some extent, perhaps further practice and more focus on word spelling and word reading would be required to allow for the development of basic word reading skills, and so, transfer effects could potentially be found later in the school year. Mavilidi and colleagues (2018) demonstrated that spelling improved following 4 weeks of 120-min intervention per week (Mavilidi et al., 2018). Therefore, one speculation of the present study is that spelling measures could potentially demonstrate intervention transfer effects. However, this type of testing was not performed and therefore remains a potential focus for future research. Intervention duration and intensity could also explain the absence of transfer effects on reading skills in the present study. Botha and colleagues (2020) demonstrated a significant effect of a visuomotor intervention on reading skills using a standardized reading test, which is similar to the reading test used in the present study (Botha & Africa, 2020). However, they used an intervention of 12 weeks with 120 min a week (in total 1440 min), which is twice the exposure time compared to the present study (8 weeks of 90 min = 720 min in total). Therefore, it could be assumed that the manifestation of transfer effects may be dependent on the dose of reading activities.

### The Relationship between Motor Skills and Letter-Sound Knowledge

In addition, we wanted to investigate the association between children’s motor skills and their pre-reading and word reading skills. We expected a significant relationship based on other studies that reported significant correlations between children’s motor skills and academic performance (Cameron et al., 2016; Geertsen et al., 2016; Murrah, 2010). The present study did not find any evidence to support this claim since no correlations were found between balance and dexterity measures and academic outcomes. Further correlational analyses were performed to see if children’s motor skills were associated with the prevalence of using movements while naming letter-sounds, but no evidence for such association was found. We did not find any evidence to suggest that children could be limited in their ability to participate in movement-based learning and thereby not benefit from the facilitative effects of this learning strategy. However, this contrasts with other findings. For instance, Botha and Africa (2020) found a significant correlation between reading skills and motor skills in a study with 6–7-year-old children. They used the short form of the Bruininks-Oseretsky Test of Motor Proficiency second edition (BOT-2) (Bruininks & Bruininks, 2005) which includes 14 items within five different movement areas (Botha & Africa, 2020), therefore providing a comprehensive assessment of individual motor proficiency. It has been suggested that different types of motor skills are associated with specific cognitive processes throughout development (Ludyga et al., 2019; Piek et al., 2008), so this motor and reading skills relationship was more likely to be detected with a more comprehensive battery of motor skill assessments. The present study only evaluated participating children’s superficial motor functioning using one gross motor skill test (flamingo balance test) and one fine motor movement test (pegboard). This was sufficient for the aims of the current study as both tests are sensitive enough to indicate significant motor difficulties. However, future research looking at the relationship between motor and reading skills should consider using more comprehensive motor skills assessments such as BOT-2 (Bruininks & Bruininks, 2005).

#### How to Use Embodied Learning in a School Setting

##### Movements vs Handwriting

In this study, movement and learning content in the two intervention groups had a close connection. Based on Skulmowski taxonomy, our whole-body movement group had a high bodily engagement and high task integration, whereas the hand movement group had a low bodily engagement and high task integration (Skulmowski & Rey, 2018). This was in contrast to our control group which did not have a high bodily engagement, and the task integration was more incidental. The control group did not combine handwriting with letter-sounds, and thereby, the integration was more incidental. Previous research has suggested that handwriting constitutes a motor-embodied learning task that is beneficial for, e.g., recognizing letters at start of literacy (James, 2017; Longcamp et al., 2005). However, our results indicate that high bodily engagement and high task integration are superior since intervention groups performed significantly better in letter-sound knowledge compared to control situation, who only performed handwriting. Thereby, we recommend that activities with a high bodily engagement and task integration are used as a supplement to handwriting when learning children’s letter-sound associations.

##### Whole-Body vs Hand Movements

Based on our results, there is no evidence to indicate that the amount of bodily engagement (using arms vs using the whole body) affected the consolidation of the letter-sound knowledge immediately after the intervention. However, the amount of bodily engagement was observed to have a long-term effect with significant differences observed following the retention period. This indicates that it is important to have high bodily engagement and task integration to improve learning efficiency for long-lasting gains in the context of letter-sound knowledge. This is in line with Skulmowski’s conclusion; studies with low bodily engagement or only incidental embodiment manipulations result in weak effects on some performances, and increasing the degree of bodily engagement and integration may in some cases lead to higher learning outcomes (Skulmowski & Rey, 2018). In connection, Craik and Lochart’s (1972) processing framework suggested that information processed deeply is easier to remember. This may be an explanation for the long-lasting effect for our whole-body movement group, where the higher level of bodily engagement may induce deeper processing benefitting memory encoding and retrieval (Madan & Singhal, 2012). However, further studies are needed to find the underlying mechanism behind this finding.

We recommend that teachers should incorporate movement-based teaching in their standard teaching curricula with specific consideration for whole-body movements. Some studies report a risk of cognitive overload with high bodily engagement, and in the end, some researchers have defined a medium degree of interactivity to be best suited for increasing learning performance. Our findings may thereby be found in both the degree of embodiment and the high task integration, without causing a cognitive overload for the children.

For interest, every activity used in the intervention can be found in our protocol article from 2021 (Gejl et al., 2021).

## Strengths and Limitations

One important strength of the study was the high level of control and randomization. Having developed teaching material based on the research-founded Danish teaching material, Fandango Mini, which is recognized and used by several preschool teachers in Denmark (Jacobsen & Veber Nielsen, 2011), we ensured that all participants were taught exactly the same content. The difference between the three groups was the degree of movement ranging from incidental handwriting to whole-body movements. The two movement groups were extremely similar and were very much alike. The difference in the delivered intervention was conceptualized by the degree of body engagement as either the hand movements (i.e., low movement range primarily using small muscles) or the whole-body movement (i.e., high movement range using small and large muscles). This presents a unique contribution of the current study to the field of embodied learning as it addresses the important distinction between the types of movements that can be applied in educational interventions, which is not commonly observed in the literature.

The activities in the two intervention (WM/HM) groups were performed by trained external instructors and by the class’ own teacher in CON. This difference could have an impact on the outcome of the study. Children in CON could have an advantage over the movement groups because they had their own teacher with whom they were more familiar. This could present a greater opportunity to achieve better outcomes following the intervention period. It was observed that some teachers in the control condition used movement themselves when teaching letter-sound correspondences. However, we did not find any effects in the knowledge of letter-sound correspondences for CON compared to the other groups which indicate the importance of children performing movement themselves instead of watching teachers performing movements.

The fidelity was evaluated by register the amount of performed exercises and compliance to the protocol was registered to ensure the quality of the intervention.

Motivation was measured during the intervention with a developed questionnaire (Gejl et al., 2021). However, the children were not able to perform it correctly, which is why data is not analyzed because of mistrust of data.

## Conclusion

The present intervention study contributes to the understanding of embodied learning and its use to improve children’s letter-sound knowledge. We conclude that there are beneficial effects of using whole-body movements for children when learning letter-sounds. In addition, hand motor movements also had an effect on letter-sound knowledge; however, the whole-body movements had longer-lasting effects.

Further intervention studies focusing on the underlying mechanisms of embodied learning effects should be investigated, and more intervention studies focusing on the degree of embodiment should be performed. From a practical perspective, teachers should consider integrating a higher degree of embodied learning activities when teaching letter-sound correspondences in early literacy instruction.
